# Placental Vacuolar ATPase Function Is a Key Link between Multiple Causes of Preeclampsia

**DOI:** 10.1155/2013/504173

**Published:** 2013-05-22

**Authors:** Dongxin Zhang, Duyun Ye, Hongxiang Chen

**Affiliations:** ^1^Department of Pathophysiology, Tongji Medical College, Huazhong University of Science and Technology, No. 13, Hangkong Road, Wuhan 430030, China; ^2^Department of Dermatology, Union Hospital, Tongji Medical College, Huazhong University of Science and Technology, Wuhan 430022, China; ^3^Department of Dermatology, Cutaneous Biology Research Center, Massachusetts General Hospital, Harvard Medical School, Boston, MA 02129, USA

## Abstract

Preeclampsia, a relatively common pregnancy disorder, is one of the major causes of maternal and fetal morbidity and mortality. Despite numerous research, the etiology of this syndrome remains not well understood as the pathogenesis of preeclampsia is complex, involving interaction between genetic, immunologic, and environmental factors. Preeclampsia, originating in placenta abnormalities, is induced by the circulating factors derived from the abnormal placenta. Recent work has identified various molecular mechanisms related to placenta development, including renin-angiotensin system, 1, 25-dihydroxyvitamin D, and lipoxin A4. Interestingly, advances suggest that vacuolar ATPase, a key molecule in placentation, is closely associated with them. Therefore, this intriguing molecule may represent an important link between various causes of preeclampsia. Here, we review that vacuolar ATPase works as a key link between multiple causes of preeclampsia and discuss the potential molecular mechanisms. The novel findings outlined in this review may provide promising explanations for the causation of preeclampsia and a rationale for future therapeutic interventions for this condition.

## 1. Preeclampsia

Preeclampsia, a pregnancy-complicated syndrome, is one of the leading causes of maternal and fetal morbidity and mortality [1]. This potentially dangerous condition affects approximately 2–7% of all pregnancies in the western world, but the incidence in developing countries can be greater [1, 2]. Preeclampsia is a maternal systemic symptom of late second or third trimester of pregnancy, characterized by hypertension, proteinuria, fetal intrauterine growth restriction, and other maternal abnormity [3]. In the worst cases, it may threaten the survival of both mother and baby [4]. Now, except for induced delivery, there is no effective treatment to cure it. Risk factors involved in preeclampsia have been increasingly identified (Table 1), and emergence of any risk factor may cause a woman to develop preeclampsia. However, exact pathogenesis of this disorder remains uncertain since numerous research into the etiology of preeclampsia did not progress significantly. Understanding the detailed mechanism underlying preeclampsia will help to develop novel prophylactic and therapeutic interventions for this condition.

The current understanding about preeclampsia is that it appears to develop in a two-stage progress, with a preclinical initial stage which has an abnormal placenta, followed by a second stage during which release of soluble factors from hypoxic placenta leads to various observed maternal conditions, including hypertension, proteinuria, and generalized damage to the maternal organ. Preeclampsia is commonly considered to result from the presence of a poor placenta since the only effective cure of the clinical syndrome is delivery of the placenta. Placentation occurs from weeks 6 to 18 of gestation, during which extravillous cytotrophoblast invasion is required for extensive remodeling of maternal spiral arteries [4]. In preeclampsia, placenta is extremely shallow because of inadequate trophoblast invasion and deficient remodeling of spiral arteries [5, 6]. Implantation failure of placenta predisposes it to hypoxia and oxidative stress [7], thus resulting in a maternal systemic inflammatory response characterized by enhanced expression of inflammatory mediators [5]. It was found that dysfunctional placenta can release adverse factors into the maternal circulation, causing the clinical features of this condition. These factors include some hormones or chemical substances, such as sFlt1 (soluble fms-like tyrosine kinase 1) [4], AT1-AA (angiotensin II AT1 receptor auto-antibody) [6], and cardiac glycosides (marinobufagenin, MBG) [8]. Among these, sFlt1 can cause endothelial dysfunction in the maternal blood vessels by binding vascular endothelial growth factors and placental growth factor [4]; AT1-AA can react with the AT1 receptor in a stimulatory fashion similar to angiotensin II signalling, which could contribute to maternal hypertension [6]; MBG, involved in the pathogenesis of preeclampsia, has been demonstrated to cause hypertension, proteinuria, and intrauterine growth restriction in the rat model of preeclampsia [8]. Augmented release of these factors secondary to the original poor placenta will eventually lead to damage of the endothelium, metabolism dysfunction, and inflammation as well as other typical symptoms. 

**Figure 1:**
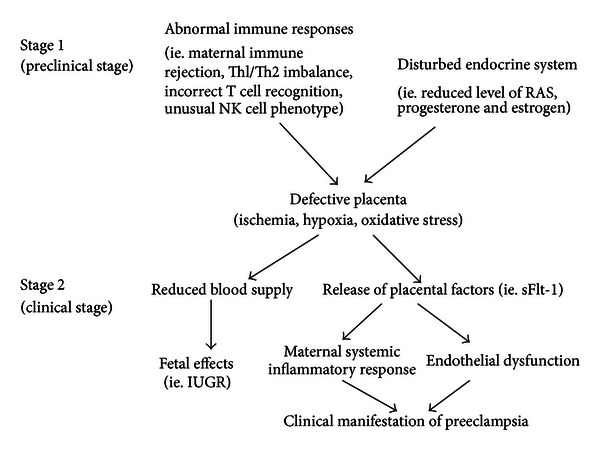
Abnormal immune responses and endocrine system in the development of preeclampsia. Preclinical stage 1 occurs in early pregnancy when abnormal immune responses and disturbed endocrine system lead to poor placentation resulting in placental hypoxia, ischemia and oxidative stress. Clinical stage 2 occurs systemically when a defective placenta releases factors into the maternal circulation, which cause the maternal systemic inflammatory response and endothelial dysfunction that lead to the clinical signs of preeclampsia.

Then, what is the exact mechanism leading to deficient trophoblast invasion and further poor placenta? Immune responses and maternal endocrine system can be considered to be two main elements involved in the development of placenta. There is evidence suggesting that shallow placenta is thought to stem from the lack of immune tolerance to pater-derived fetal antigens [9]. Initial maternal rejection to the fetoplacenta may be the main reason for inadequate spiral arteries remodeling in preeclampsia. During the normal pregnancy, maternal innate immune response is physiologically regulated to prevent the rejection to the fetal allograft. Meanwhile, Th1/Th2 activity balance is strongly shifted toward Th2 activity to maintain the normal fetomaternal interaction [10]. Many have found that maternal T cell cannot recognize fetal human lymphocyte antigens (HLAs) rightly and decidua NK cell has an unusual phenotype in preeclampsia [4]. Moreover, another important difference found between normal pregnancy and preeclampsia is the shift of Th1/Th2 balance toward Th1 responses. In addition to immune maladaptation, it is also found that maternal endocrine system is perturbed in preeclampsia. In normal pregnancy, components of renin-angiotensin-aldosterone system, including renin, angiotensin, and aldosterone, are increased, while these hormones are suppressed in preeclampsia [9]. A recent report found that activation of mineralocorticoid receptor in trophoblast by maternal aldosterone appears to be required for trophoblast proliferation and placenta development [11]. Additionally, other pregnancy-related hormones also make great contribution to favourable pregnancy outcome. For example, progesterone plays a dominant role in maintaining uterine quiescence and modulating fetomaternal immune response locally, while estrogen appears also to be a potent immunomodulator in pregnancy-associated immune function [10]. Aberration in these hormones level may disrupt homoeostasis of placenta development, thus causing the onset of preeclampsia eventually. Overall, abnormal change in immune responses and maternal endocrine system throughout pregnancy may be one explanation for preeclampsia (Figure 1). 

Following extensive research into placentation, recent advances have identified emerging key players relevant to placenta establishment, including renin-angiotensin system (RAS), 1, 25-dihydroxyvitamin D (1,25(OH)2D3), and lipoxin A4. Evidence indicates that aberration in them may disrupt placenta development, contributing to adverse pregnancy outcome, such as preeclampsia (described subsequently). V-ATPase, another key molecule in placentation, is closely associated with all of them. During normal pregnancy, RAS, 1,25(OH)2D3, and lipoxin A4 may work independently, in parallel or jointly on the central molecule, V-ATPase, while V-ATPase also affects them at the same time. Therefore, the intriguing molecule V-ATPase may represent a key link between various causes of preeclampsia. In the following the present review will focus on some new findings about interaction between V-ATPase and RAS, 1,25(OH)2D3 and lipoxin A4 in pregnancy, which will provide new insights into the molecular mechanisms underlying preeclampsia.

## 2. Vacuolar ATPase

The acidity regulation of intracellular and extracellular environment is crucial for a variety of biological processes, including acidity homeostasis, membrane trafficking, protein degradation, and sperm maturation [14]. Central to this regulation is the Vacuolar ATPase (V-ATPase), which is a large multisubunit complex functioning as an ATP-driven proton pump. V-ATPase is of significant importance in basic physiological processes, and it has been implicated in a number of human diseases, such as renal disease, tumour metastasis, and bone disease [14]. V-ATPase is composed of two large domains, V0 and V1. The V0 domain is a 260 kDa membrane-embedded complex that contains six different subunits (a, d, e, c, c′, and c′′) and functions as a proton-translocation domain, whereas the V1 domain is a 650 kDa cytoplasmic complex that contains eight different subunits (A, B, C, D, E, F, G, and H) and functions as an ATP-hydrolytic domain. It is the structural complexity of V-ATPase that makes it an important player in various physiological functions. Many of V-ATPase subunits have multiple isoforms, and so specific V-ATPase can be composed of different combinations of these subunit isoforms. 

**Figure 2:**
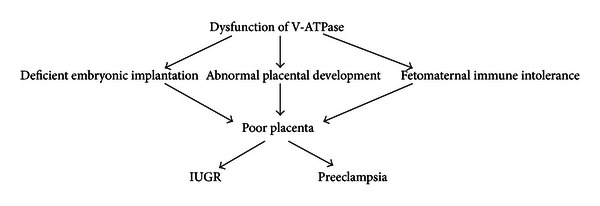
Abnormal V-ATPase function in the development of preeclampsia. Preclinical stage of preeclampsia occurs in early pregnancy when dysfunctional V-ATPase activity leads to deficient embryonic implantation, abnormal placental development and fetomaternal immune intolerance, resulting in poor placenta. Clinical stage of preeclampsia occurs systemically when a defective placenta causes the maternal systemic inflammatory response and endothelial dysfunction that lead to the clinical signs of preeclampsia.

V-ATPase, mediating various physiological processes and cellular homeostasis, may be closely involved in different stages of reproduction. In fact, there are many reports demonstrating that V-ATPase has an important role in all aspects of mammalian reproduction, such as spermatogenesis, fertilization, embryonic implantation, and embryo development [15]. On one hand, the acidic microenvironment regulated by V-ATPase is essential for embryonic development following embryonic implantation. On the other hand, different V-ATPase has specific tissue distribution in reproduction system and exerts differential crucial function. Among all of the V-ATPase subunits, subunit “a” is involved in acrosomal reaction of sperm and embryonic implantation, both of which provide vital foundation for successful pregnancy [15]. Subunit “a” is a large membrane-spanning protein with a hydrophilic cytoplasmic N-terminal domain and a nine-transmembrane C-terminal domain [14], and it contains the information necessary to target the V-ATPase to different cellular destinations and is responsible for proton transport into the lumen of intracellular vesicles or across membranes [16]. There are four isoforms of subunit “a” (a1, a2, a3, and a4), which exhibit diverse tissue distribution and subcellular location [16]. Isoform 2 of subunit “a” (ATP6V0A2, referred to as a2V-ATPase) can be detected in the epididymis, kidney, lung, thymus, spleen, and placenta [17]. Recently, increasing evidence indicates the crucial role of a2V-ATPase in placental immune modulation associated with pregnancy [15]. In early pregnancy, many important cytokines secreted by placenta trophoblast cells and immune cells in decidua play a key role in embryonic implantation and fetomaternal immune tolerance [18]. Interestingly, it is reported that a2V-ATPase is important in controlling the expression of these essential cytokines [18]. Thus, the normal expression and localization of a2V-ATPase in fetoplacental unit is essential for pregnancy. Jaiswal et al. have reported that placental a2V-ATPase expression is a link between multiple causes of spontaneous abortion in mice, and dysfunction of a2V-ATPase in early pregnancy has been associated not only with spontaneous abortion but also with preeclampsia and fetal growth restriction [19]. Another interesting study found that NOX2/NADPH oxidase regulates antigen crosspresentation in adaptive immunity through changing phagosome lumen PH, whereas the effect of NOX2/NADPH oxidase on phagosomal pH is antagonized by V-ATPase, which may represent a novel mechanism responsible for the maintenance of immune tolerance [20]. Taken together, these indicate the importance of V-ATPase in maintaining reproduction process and placentation and modulating fetomaternal immune tolerance in pregnancy, whereas defective V-ATPase may increase the risk of causing a poor placenta and inducing preeclampsia (Figure 2). 

## 3. Renin-Angiotensin System, V-ATPase, and Preeclampsia

The circulating renin-angiotensin system (RAS) is an endocrine hormone system that plays a key role in regulating blood pressure and systemic electrolyte balance. Apart from systemic circulating RAS, local RAS is present in many tissues or organs, such as brain, heart, ovary, and placenta, and one of the major extrarenal RAS during pregnancy is the placental RAS [21]. In pregnancy, the circulating RAS maintains maternal electrolyte balance and blood pressure homoeostasis, facilitating expanding blood supply for fetus, while the placental RAS has indispensable role in the uteroplacental unit [21]. Components of placental RAS have been confirmed to be involved in many molecular events of placental establishment. A recent review reported that placental cytotrophoblast is rich in AT1 receptor, and ANG II-AT1 receptor signaling regulates multiple genes associated with normal trophoblast invasion, such as plasminogen activator inhibitor-1 (PAI-1), such as soluble fms-like tyrosine receptor-1 (sFlt-1) and soluble endoglin (s-Eng) [22]. Additionally, ANG II signaling also activates NF-kappa B and stimulates the synthesis of reduced nicotinamide-adenine dinucleotide phosphate (NADPH) oxidase by trophoblast [22]. Moreover, it has been demonstrated by a recent study that activation of mineralocorticoid receptor in placental trophoblast by maternal aldosterone appears to be required for trophoblast growth and normal fetoplacental function [11]. Therefore, in response to pregnancy, both systemic RAS and placental RAS need to be adapted to sustain a healthy pregnancy. 

Advances have revealed that maternal RAS is perturbed in preeclampsia. In an uncomplicated pregnancy, there is an increase in almost all the components of maternal RAS, including renin, angiotensin, and aldosterone, but these hormones are suppressed in preeclampsia [9]. However, the changes happening in the components of uteroplacental RAS are different from those observed in circulating RAS. Recent studies demonstrated that upregulation of the AT1 receptor expression [23], increase in renin level [24], and enhancement of ANG II production [25] occur in uteroplacental unit of preeclamptic women. So, the expression profile of both systemic RAS and placental RAS in preeclampsia differs greatly from that of healthy uncomplicated pregnancy. Additionally, there is a growing body of reports indicating the presence of the angiotensin II type I receptor agonistic autoantibody (AT1-AA) in preeclamptic women, and this autoantibody through AT1 receptor signaling can result in dysregulation of the RAS, then leading to clinical features of preeclampsia [22]. All abnormalities in both systemic RAS and placental RAS can cause poor placentation and preeclampsia.

There is accumulating evidence suggesting that components of RAS are interrelated to V-ATPase and are essential for regulating V-ATPase activity. The (pro)renin receptor ((P)RR), encoded in *ATP6AP2*, plays a key role in activation of the local RAS, while a truncated form of (P)RR, termed M8.9, was found to be associated with the V-ATPase assembly and function, implicating a novel role of (P)RR in V-ATPase activity [26]. Recently, another interesting report indicated that (P)RR functions as an adaptor between Wnt receptor and V-ATPase, which is required for mediating Wnt signaling [27]. It was found that angiotensin II has profound effects on H^+^ transport and urinary acidification in kidney, mainly through its regulation on V-ATPase activity, and it has also been confirmed to stimulate vacuolar H^+^-ATPase activity in renal acid-secretory intercalated cells from the outer medullary collecting duct [28]. Not long before, a report showed that aldosterone may participate in the regulation of V-ATPase activity in acid-secretory intercalated cells through rapid nongenomic stimulatory action and contribute to urinary acidification [29]. Meanwhile, another study, prior to this review, described that aldosterone has both genomic and nongenomic stimulatory effects on V-ATPase in proximal S3 segments [30]. Considering the content described earlier, it is reasonable that uteroplacental V-ATPase function is a key link between RAS and placentation. Therefore, any aberration in interaction between RAS and V-ATPase may induce preeclampsia by interfering various processes in placent development.

## 4. 1,25-Dihydroxyvitamin D, V-ATPase, and Preeclampsia

1,25-Dihydroxyvitamin D (1,25(OH)2D3), the active form of vitamin D, has long been considered to be a leading molecule in calcium and bone homeostasis. However, in the past years, it was found that vitamin D receptor (VDR) is present in most tissues and many tissues contain the 1,25(OH)2D3 synthase, 25(OH)D3-1*α*-hydroxylase [31, 32], which makes us begin to be aware of multiple nonclassic actions of 1,25(OH)2D3. In fact, advances have shown that 1,25(OH)2D3 deficiency may increase the risk of developing a wide range of chronic diseases, including cancer, autoimmune disease, infectious disease, and cardiovascular disease [32]. Through binding to the VDR, 1,25(OH)2D3 can generate a wide array of favorable nonclassic biological responses that are different from its classic actions in maintaining bone and calcium homeostasis, whereas these nonclassic actions are mainly linked to its regulation of cell proliferation, cell differentiation, and immune responses [31]. It is worth emphasizing that some of nonclassic biological effects of 1,25(OH)2D3 have been suggested to participate in modulation of embryo implantation, fetomaternal immune tolerance, and placental antimicrobial and anti-inflammatory responses [33]. Therefore, 1,25(OH)2D3 deficiency in pregnancy may not only impair maternal and fetal bone health, but also cause various adverse pregnancy outcomes such as preterm birth, fetal intrauterine growth restriction, and preeclampsia. Indeed, a clinical study by Halhali et. al demonstrated that the level of 1,25(OH)2D3 in both maternal circulation and umbilical cord compartment is low in preeclampsia [34]. Furthermore, expression and activity of 25-hydroxyvitamin D-1*α*-hydroxylase has been confirmed to be significantly restricted in cultures of placental syncytiotrophoblast cells from preeclamptic pregnancy [35]. So, 1,25(OH)2D3 insufficiency during pregnancy is potentially associated with increased risk of preeclampsia.

Typical feature of preeclampsia is maternal systemic inflammatory response, characterized by upregulation of proinflammatory mediators in both uteroplacental unit and peripheral circulation. In fact, as described previously, the systemic inflammatory response in preeclampsia is thought to stem from initial disorder of fetomaternal immune tolerance. Vitamin D, particularly 1,25(OH)2D3, has received particular attention in recent years as it has been shown to have important effect on both innate and adaptive immune systems. Moreover, 1,25(OH)2D3 has been demonstrated to be essential for modulating tissue-specific immune responses and preventing chronic inflammation and autoimmunity [36]. Increasingly, the immunomodulation of 1,25(OH)2D3 has been proposed as a key link between vitamin D and pregnancy [33]. In addition to be mainly synthesized in the kidney, 1,25(OH)2D3 can also be locally produced by immune cells, such as macrophage, dendritic cell, activated T cell, and probably B cell [36]. In autocrine or paracrine manner, the local produced 1,25(OH)2D3 can exert a marked inhibitory effect on adaptive immune responses, promoting humoral Th2 responses whilst suppressing cellular Th1 responses [33, 36]. Also, innate immune cells can be inhibited by 1,25(OH)2D3. For example, 1,25(OH)2D3 is able to inhibit the differentiation, maturation, and immunostimulatory capacity of DCs by decreasing the expression of MHC class II molecule, CD40, CD80, and CD86 [36]. A recent report by Saito speculated that imbalance between regulatory T cell and Th17 cell differentiation may explain pathogenesis of preeclampsia [37], which provides new light on pathophysiology of preeclampsia. Interestingly, 1,25(OH)2D3 has been confirmed to enhance tolerogenic immunity by inducing immunosuppressive Treg [38], while proinflammatory Th17 cell is suppressed by 1,25(OH)2D3 [39]. In summary, maternal vitamin D level may influence pregnancy outcome through a variety of potential mechanisms, and impaired vitamin D status may lead to preeclampsia. 

There is an intimate interaction between vitamin D and V-ATPase. Indeed, a recent study demonstrated that in the presence of vitamin D, human osteoclasts express elevated V-ATPase levels [40]. Meanwhile, this work also described that 1,25(OH)2D3 can upregulate the activity of V-ATPase complex [40]. Although the study did not study the case in pregnancy, it will imply us that 1,25(OH)2D3 may have an important effect on uteroplacental V-ATPase activity. Moreover, the ability of 1,25(OH)2D3 to regulate secretion of pregnancy-related hormones may explain its role in mediating V-ATPase activity. 1,25(OH)2D3 has been confirmed to be an important player in the upregulation of estrogen biosynthesis [41], and the previous description has also revealed that V-ATPase expression is estrogen dependent [42], which makes it reasonable to speculate that 1,25(OH)2D3 can act on V-ATPase indirectly by influencing hormones level. Recently, a significative review suggested that autophagy is a general basis for the multiple health-promoting effects of vitamin D [43]. Autophagy, depending on lysosomal self-digestion machinery, is an evolutionarily conserved self-catabolism process that is essential for cell growth, development, and homeostasis. To a great extent, V-ATPase is vital for autophagy since V-ATPase is of great importance for lysosomal function. Thus, coupled with V-ATPase, 1,25(OH)2D3 could induce and activate autophagy, protecting human against many diseases. Autophagy is induced by several forms of cell stress including hypoxia, infection, and starvation [43]. If autophagy could not be initiated properly, disturbance in autophagy will result in a wide range of disorder. In the case of preeclampsia, abnormal placentation in early pregnancy predisposes placenta to ischemia, hypoxia, and oxidative stress, which accumulates large numbers of apoptotic trophoblast cells at uteroplacental interface. If not treated through cell autophagy, the apoptotic trophoblast cells may impair further placenta development seriously and induce onset of preeclampsia. Thus, maternal vitamin D deficiency may trigger preeclampsia through affecting V-ATPase activity, which seems to represent a general and basic pathogenesis of preeclampsia.

## 5. Lipoxin A4, V-ATPase, and Preeclampsia

Lipoxin, the “stop signal” for inflammation, is the first mediator recognized to have dual anti-inflammatory and proresolution activities [44]. In mammals, lipoxin A4 is the major lipoxin that has potent actions *in vivo*. Lipoxin A4, mainly depending on transcellular biosynthesis in the inflammation microenvironment, is derived from arachidonic acid by the sequential action of lipoxygenase (LOX). Leukocyte/platelet interaction, involving two key enzymes 5-LOX and 12-LOX, is one of the most important synthesis forms. This pattern of biosynthesis involves the initiation of arachidonic acid oxygenation by 5-LOX in leukocytes, and then the intermediate leukotriene A4 is converted into lipoxin A4 by 12-LOX in platelets. Interestingly, our previous results indicated that platelet-derived microparticles containing 12-LOX can be transferred to leukotriene-producing mast cells, which represents a new pathway for lipoxin A4 biosynthesis* in vivo* [45]. Lipoxin A4 can act on many cell types including blood cell, neural cell, and stromal cell [44]. Lipoxin A4 displays selective regulation for leukocyte responses and trafficking *in vivo *through activating its specific receptor, ALXR. For example, in neutrophils, lipoxin A4-ALXR interaction blocks neutrophil migration and infiltration into sites of inflammation. By contrast, in monocytes, LXA4-ALXR interaction stimulates monocyte recruitment and the phagocytosis of apoptotic neutrophils. Recently, we found that lipoxin A4 can suppress hepatocellular carcinoma via remodeling tumor microenvironment [46]. Moreover, we found that ALXR (also termed FPR2) has an appreciable pleiotropic regulator role in macrophage polarization, and LXA4-induced M2a+M2c-like macrophage phenotype showed antitumorigenesis activities [47]. Taken together, all previous results show us the key role of lipoxin A4 as an essential modulator of immune response and homoeostasis.

Inflammatory processes are central to a series of pregnancy events, while inflammation dysregulation is a typical feature of pregnant complications, such as preeclampsia and preterm. Normal pregnancy is characterised by a state of mild maternal systemic inflammation, which develops when pregnancy is progressing [5]. Although preeclampsia is also associated with inflammatory response, it is an exaggerated maternal systemic inflammatory response [6]. In fact, this disorder is an extreme condition of a range of uncontrolled inflammatory responses induced by abnormal pregnancy. Lipoxin A4, as an anti-inflammatory and proresolution mediator, will play an important role in human pregnancy. In fact, level of lipoxin A4 in nonpregnant women is significantly lower than that in pregnant women, and circulating level of lipoxin A4 in pregnant women displayed a modest but significant increase throughout pregnancy [48]. Thus, it is tempting to speculate that inadequate production of lipoxin A4 can lead to abnormality of placental immunomicroenviroment, increasing the risk of preeclampsia. This has been confirmed by our results from clinical study that level of lipoxin A4 in preeclampsia is significantly lower than that in normal pregnancy. Additionally, our results from animal study demonstrated that lipoxin A4 could suppress the increased production of proinflammatory cytokines and alleviate the symptoms of preeclampsia in LPS-induced rat model for human preeclampsia [49]. Moreover, results from our previous study verified that lipoxin A4 can inhibit the enhanced IL-1*β* production of monocytes from severe preeclampsia women [50]. Recently, preliminary results from animal study indicated that lipoxin A4 may participate in the regulation of rat fetomaternal immune tolerance in early pregnancy. Therefore, lipoxin A4, an endogenous anti-inflammatory and proresolution mediator, is a key player in terminating exaggerated inflammatory responses and restoring maternal systemic homoeostasis in pregnancy. 

There exists an interrelationship between lipoxin A4 and V-ATPase. In fact, this is the case. The recent findings by Russell et al. found that lipoxin A4 is a novel estrogen receptor modulator and exhibits estrogenic activity *in vivo*, revealing a previously unappreciated facet of various bioactions of this anti-inflammatory and pro-resolution mediator [51]. Thus, lipoxin A4 may exert an important role in regulating the V-ATPase expression, as V-ATPase expression level is estrogen dependent, which has been mentioned earlier in this review. In addition, V-ATPase may also participate in the modulation of lipoxin A4 bioactions. There is evidence implicating that V-ATPase may get involved in the complex interaction between lipoxin A4 and its receptor in lipoxin A4-stimulated signal pathways. Maderna et al. reported that internalization and trafficking is critical for LXA4-stimulated phagocytosis in the resolution of inflammation [52]. Transmembrane receptors internalization is of physiological importance, which is required for right signal transduction, proper dissociation of ligand from receptor, and receptor-mediated endocytosis. Although receptor internalization may occur in different ways, V-ATPase-mediated intraendosomal pathway is the main endocytic trafficking machinery [53]. Therefore, internalization and trafficking of lipoxin A4 receptor is likely to require the right assembly and activity of V-ATPase in the endocytic complex of ALXR internalization. If there is some defect in V-ATPase, biological actions of LXA4 through ALXR could be severely hindered. In summary, lipoxin A4 may play an important role in modulating V-ATPase activity during pregnancy while the key role of lipoxin A4 in fetomaternal immunoregulation also depends on normal V-ATPase activity extensively.

## 6. Proposed Model and Conclusion

**Figure 3:**
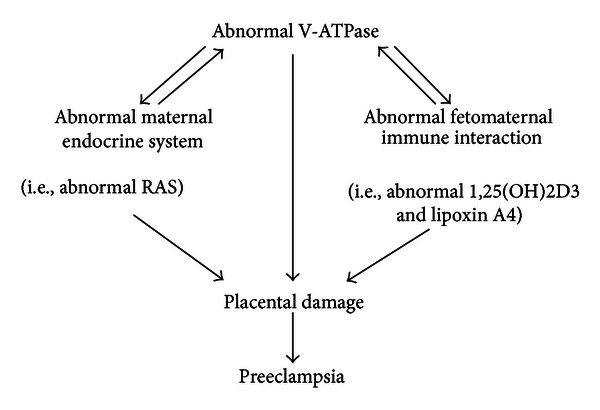
A proposed model that placental V-ATPase function is a key link between multiple causes of preeclampsia.

Based on the previous novel findings, a model of how placental V-ATPase function can be a key link between multiple causes of preeclampsia is proposed (Figure 3). On one hand, V-ATPase is an essential modulator in regulating bioaction of RAS, 1,25(OH)2D3, and lipoxin A4 during pregnancy. On the other hand, key roles of RAS, 1,25(OH)2D3, and lipoxin A4 can be integrated into the V-ATPase function in pregnancy. Right cross-talk between V-ATPase and RAS, 1,25(OH)2D3, and lipoxin A4 can facilitate normal placentation and ensure successful pregnancy. Once disorder of V-ATPase activity takes place at the fetomaternal interface, various bioactivity of RAS, 1,25(OH)2D3, and lipoxin A4 will come into great trouble, and vice versa. Any abnormality occurring at the interaction between V-ATPase and RAS, 1,25(OH)2D3 and lipoxin A4 could cause a defective placenta, which will be eventually involved in the progression of preeclampsia. Overall, the previous proposal not only suggests the importance of V-ATPase in modulating essential components of maternal endocrine system (RAS) and key molecules of fetomaternal immune interaction (1,25(OH)2D3 and lipoxin A4), but also shows the key role of RAS, 1,25(OH)2D3, and lipoxin A4 in regulating V-ATPase function. 

Almost all cases of preeclampsia are associated with poor placentation. Maternal endocrine system and immune responses have been long considered to be involved in normal placenta development. The renin-angiotensin system is a typical endocrine hormone system that plays a key role in regulating placentation as well as maintaining normal pregnancy. Recently, the immunomodulation of vitamin D in pregnancy has been proposed as a key aspect of placental homoeostasis. Additionally, lipoxin A4, as an anti-inflammatory and proresolution mediator, has been demonstrated to be vital in modulating fetomaternal immune tolerance during pregnancy. Nevertheless, the complexity of various molecular events that occur at the fetomaternal interface in placenta development is far from being completely elucidated. A series of molecular mechanisms needs to be coordinated for establishing a perfect placenta, while any deviation from this ordered course of molecular events may result in pregnancy complications, such as preeclampsia. V-ATPase, a key molecule in placentation, is beginning to be appreciated. It is closely linked to renin-angiotensin system, 1,25(OH)2D3, and lipoxin A4. Therefore, it may work as a key link between multiple causes of preeclampsia. Further studies regarding V-ATPase-related molecular mechanisms necessary for placentation are required, since clinical intervention to these potential dysregulated molecular events may eventually improve pregnancy outcome.

### 6.1. Further Research Directions

1. Determe the detailed role of V-ATPase in fetoplacental unit.
2. Clarify the potential role of placental V-ATPase in preeclampsia.
3. Investigate the nature of interaction between V-ATPase and RAS, 1,25(OH)2D3, and lipoxin A4 in normal pregnancy.
4. Investigate the causes of abnormal interaction between V-ATPase and RAS, 1,25(OH)2D3, and lipoxin A4 in preeclampsia.
5. Understand the mechanisms of placental damage associated with defective V-ATPase, RAS, 1,25(OH)2D3 and lipoxin A4 in preeclampsia.
6. Study the effect of potential therapeutic agents on placental damage and maternal clinical signs of preeclampsia.

## Figures and Tables

**Table 1:** Risk factors that may induce development of preeclampsia.

Related category	Risk factors
Genetic factors	Individual genetic susceptibility [1]
	Polymorphisms of specific genes [1]
	Preeclampsia history [12]
	Family history [12]
Immunologic factors	Paternal alloantigens [4]
Maternal pre-existing factors	Autoimmune conditions [12]
	Hypertension [12]
	Diabetes [12]
	Renal disease [12]
	Obesity [12]
Other factors	Malnutrition [13]
	Primiparity [12]
	Multiple pregnancies [12]
	Maternal age [12]
